# Hygienic Value of Mirth

**Published:** 1881-08

**Authors:** Felix L. Oswald


### Hygienic Value of Mirth.

But, aside from all this, mirth has an hygienic value that can hardly be overrated while our social life remains what the slavery of vices and dogmas has made it. Joy has been called the sunshine of the heart, yet the same sun that calls forth the flowers of a plant is also needed to expand its leaves and ripen its fruits; and without the stimulus of exhilarating pastimes perfect bodily health is as impossible as moral and mental vigor. And, as sure as a succession of uniform crops will exhaust the best soil, the daily repetition of a monotonous occupation will wear out the best man. Body and mind require an occasional change of employment, or else a liberal supply of fertilizing recreations, and this requirement is a factor whose omission often foils the arithmetic of our political economists.

To the creatures of the wildness affliction comes generally in the forms of impending danger—famine or persistent persecution; and under such circumstances the modifications of the vital process seem to operate against its long continuance; well-wishing Nature sees her purpose defeated, and the vital energy flags, the sap of life runs to seed. On the same principal an existence of joyless drudgery seems to drain the springs of health, even at an age when they can draw upon the largest inner resources; hope, too often baffled, at last withdraws her aid; the tongue may be attuned to canting hymns of consolation, but the heart can not be deceived, and with its sinking pulse the strength of life ebbs away. Nine tenths of our city children are literally starving for lack of recreation; not the means of life, but its object, civilization has defrauded them of; they feel a want which bread can only aggravate, for only hunger helps them to forget the misery *ennui.* Their pallor is the sallow hue of a cellar-plant; they would be healthier if they were happier. I would undertake to cure a sickly child with fun and rye-bread sooner than with tidbits and tedium.—DR. FELIX L. OSWALD, *in Popular Science Monthly for August.*

